# The cytokine network in acute myeloid leukemia

**DOI:** 10.3389/fimmu.2022.1000996

**Published:** 2022-09-28

**Authors:** Michela Luciano, Peter W. Krenn, Jutta Horejs-Hoeck

**Affiliations:** ^1^ Department of Biosciences and Medical Biology, Paris Lodron University of Salzburg, Salzburg, Austria; ^2^ Cancer Cluster Salzburg, Salzburg, Austria

**Keywords:** acute myeloid leukemia, cytokine signaling, inflammation, tumor microenvironment, cytokine inhibitors

## Abstract

Acute myeloid leukemia (AML) is a highly heterogeneous malignancy of the blood and bone marrow, characterized by clonal expansion of myeloid stem and progenitor cells and rapid disease progression. Chemotherapy has been the first-line treatment for AML for more than 30 years. Application of recent high-throughput next-generation sequencing technologies has revealed significant molecular heterogeneity to AML, which in turn has motivated efforts to develop new, targeted therapies. However, due to the high complexity of this disease, including multiple driver mutations and the coexistence of multiple competing tumorigenic clones, the successful incorporation of these new agents into clinical practice remains challenging. These continuing difficulties call for the identification of innovative therapeutic approaches that are effective for a larger cohort of AML patients. Recent studies suggest that chronic immune stimulation and aberrant cytokine signaling act as triggers for AML initiation and progression, facets of the disease which might be exploited as promising targets in AML treatment. However, despite the greater appreciation of cytokine profiles in AML, the exact functions of cytokines in AML pathogenesis are not fully understood. Therefore, unravelling the molecular basis of the complex cytokine networks in AML is a prerequisite to develop new therapeutic alternatives based on targeting cytokines and their receptors.

## Introduction

Acute Myeloid Leukemia (AML) is a highly aggressive and heterogenous hematological cancer characterized by the accumulation of molecular and cytogenetic mutations within hematopoietic stem and/or progenitor cells (HSPCs), leading to the establishment of leukemic stem cells (LSCs). LSCs are the source of immature myeloid progenitor cells, so-called myeloblasts or leukemic blasts, which accumulate in the bone marrow (BM), displace normal HSPCs, impair normal hematopoiesis, and eventually spread into the peripheral blood (PB), lymph nodes, liver, spleen, testes, and central nervous system (1–4). Whereas many AML patients follow an aggressive clinical course with an overall 5-year survival rate of only 28%, individual patient survival strongly depends on the underlying tumor-driving genetic alterations and individual risk factors, including age, gender, prior chemotherapy, radiation exposure and genetic predisposition (1, 2, 5–8). However, irrespective of the molecular driver mutations initiating the disease, AML onset and development always go hand in hand with significant remodeling of the BM into a tumor-promoting microenvironment that supports and protects LSCs at the expense of normal HSPCs (9–15). In this review we discuss how cytokine signaling networks contribute to these maladaptations, fuel AML tumorigenesis and progression, and enable chemoresistance and immune evasion. We further shed some light on promising therapeutic approaches targeting cytokine signaling to irradicate the LSC population and prevent relapse after chemotherapy.

## Role of cytokines in AML

Within the healthy BM microenvironment, hematopoietic stem cells (HSCs) are normally maintained in a delicate balance between quiescence, self-renewal, and differentiation to ensure life-long steady-state hematopoiesis and replenishment of the blood effector cell population under stress conditions such as infection, acute and chronic inflammation, aging or bleeding (16). During infection and inflammation, an array of cytokines, including Interleukin (IL)-1β (17, 18), IL-3 (19, 20), IL-6 (21–23), Tumor necrosis factor-α (TNF-α) (24–27) and Interferon (IFN) (28, 29) together with hematopoietic growth factors (HGFs) such as M-CSF, G-CSF and GM-CSF (17, 30), orchestrates the switch from steady-state to emergency hematopoiesis (31–33). In patients with preleukemic and leukemic conditions, including AML, the tight regulation of these cytokines is impaired, leading to aberrant cytokine secretion (32–38). Studies evaluating pro- and anti-inflammatory cytokine and growth factor levels in serum revealed that GM-CSF, IL-1β, IL-3, IL-4, IL-5, IL-6, IL-8, IL-10, IL-12p70, IL-27, IL-35, osteopontin and stem cell factor (SCF) are upregulated in all or in distinct AML patient groups compared to age-matched controls (39–43). In contrast to most cytokines, TRAIL and TGF-β levels are decreased in the serum of AML patients (39, 43, 44). To gain insights into the specific functions of individual cytokines and growth factors in AML, numerous studies have characterized the effects of recombinant cytokines and HGFs on proliferation and colony formation of primary AML cells and cell lines *in vitro*, thereby establishing *ex vivo* AML cell culture conditions (see **Table 1**).

**Table 1:** Cytokines and growth factors supporting or inhibiting AML cells.

Cytokine	Expression in AML patients compared to healthy individuals	Physiologic function	Function *ex vivo* in AML cell culture
**G-CSF**	Not determined	Hematopoietic growth factor	Supports AML cell proliferation and clonogenicity (45–48)
**GM-CSF**	Elevated PB plasma levels and unchanged BM levels (42, 49)	Hematopoietic growth factor	Supports AML cell growth and self-renewal (44, 45, 50)
**IFN-α**	Not determined	Anti-/Pro-inflammatory cytokine	Reduces AML cell proliferation and IL-1, IL-6, GM-CSF expression (51, 52)
**IFN-γ**	Unchanged PB levels and reduced BM levels (39, 53)	Pro-inflammatory cytokine	Reduces AML cell proliferation and survival; increases spontaneous clonogenicity of AML cells (54, 55)
**IL-1Ra**	Elevated PB and reduced BM serum levels (42, 56)	Anti-inflammatory cytokine	Reduces AML cell proliferation (57, 58)
**IL-1β**	Unchanged or elevated PB and unchanged BM levels (39, 41, 42)	Pro-inflammatory cytokine	Supports AML cell proliferation and survival; increases GM-CSF, IL-6 and TNF expression (41, 45, 50, 51, 59, 60)
**IL-3**	Elevated PB levels (43)	Pro-inflammatory cytokine	Supports AML cell proliferation and self-renewal (45, 47, 61–63)
**IL-4**	Elevated PB levels in patients > 65 years (39, 53)	Anti-inflammatory cytokine	Inhibits IL-1- and HGF-induced AML cell proliferation (60, 64, 65)
**IL-6**	Elevated plasma levels (39, 53, 66)	Pro-inflammatory cytokine	Partially supports AML cell proliferation (45, 48, 67–71).
**IL-8**	Elevated PB and BM levels (39, 44, 66, 72)	Chemoattractant cytokine (chemokine)	Not determined
**IL-10**	Elevated PB levels (39, 53, 56, 73)	Anti-inflammatory cytokine	Inhibits AML cell proliferation; reduces IL-1α, IL-1β, IL-6, GM-CSF and TNF-α expression (74–76)
**IL-12p70**	Elevated PB levels in patients > 65 years (39)	Pro-inflammatory cytokine	Inhibits AML cell-induced angiogenesis; supports T cell-mediated cytotoxicity and possibly AML tumor growth (77–79)
**IL-27**	Elevated PB and BM levels (40)	Anti-inflammatory cytokine	Not determined
**IL-35**	Elevated PB and BM levels (40, 80, 81)	Anti-inflammatory cytokine	Supports AML cell proliferation and survival; promotes Treg function (80)
**Osteopontin**	Elevated PB and BM levels (44, 82, 83)	Matrix glycoprotein with pro-inflammatory cytokine properties	Supports AML cell self-renewal, proliferation and survival (84)
**SCF**	Elevated PB and BM levels (42)	Hematopoietic growth factor	Supports AML cell proliferation and survival (85–87)
**TGF-β**	Reduced PB and BM levels (39, 53)	Anti-inflammatory cytokine	Inhibits AML cell proliferation and survival (88–90)
**TNF-α**	Elevated PB levels (39, 44, 56, 66)	Pro-inflammatory cytokine	Supports AML cell chemoresistance and maintains proliferating LSCs (91)
**TRAIL**	Reduced PB levels (44)	Pro-inflammatory cytokine	Not determined
**CXCL12**	Reduced expression in AML blasts (92–94)	Chemoattractant cytokine (chemokine)	Promotes AML cell growth, survival, chemoresistance and adhesion (95–98)

Importantly, not all patient-derived AML cells or cell lines respond to HGF and cytokine treatment equally well. These observations reflect AML heterogeneity and suggest the presence of leukemic cell subpopulations. PB: peripheral blood; BM: bone marrow.

**Figure 1:**
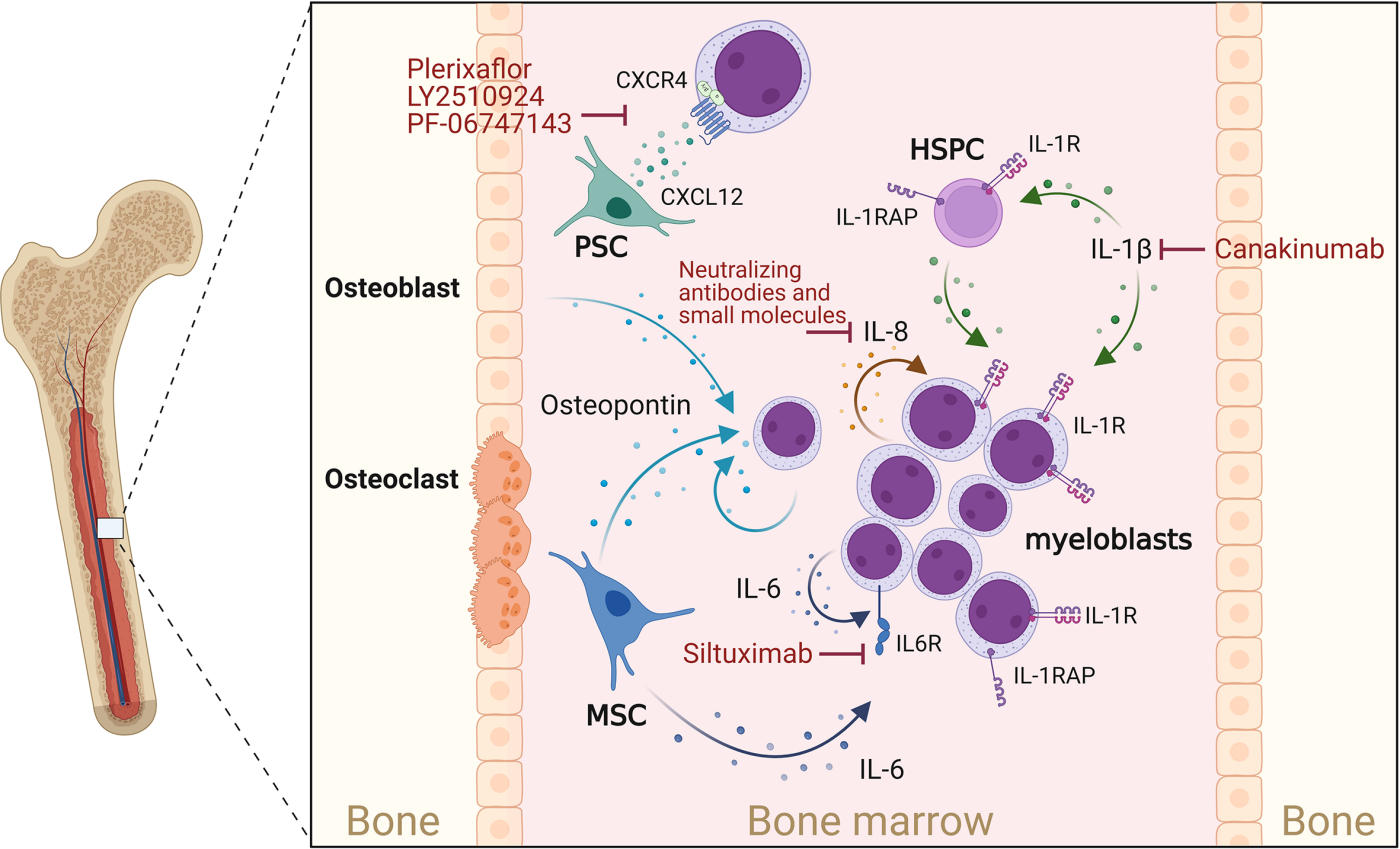
Cytokines supporting AML progression. Osteoblasts, myeloblasts and mesenchymal stromal cells (MSCs) secrete osteopontin. This in turn promotes AML cell proliferation and disease progression. CXCL12 is mainly secreted by perivascular stromal cells (PSCs), and osteoblasts and promotes growth and survival of AML blasts cell *via* the chemokine receptor CXCR4. IL-1β acts on myeloblasts and HSPCs, which express the IL-1 receptor (IL-1R) as well as the IL-1 receptor accessor protein (IL-1RAP), thereby enhancing IL-1β production, AML cell proliferation and survival. IL-1 signaling can be blocked by Canakinumab, a human monoclonal antibody targeting IL-1β. IL-8 is constitutively produced by AML myeloblasts and acts in an autocrine way. MSCs and myeloblasts are potent sources of IL-6, which can be blocked by IL-6-blocking antibodies such as Siltuximab. Created with Biorender.com.

Suggestive of a supportive feedback loop, some patient-derived LSCs and blasts can produce a variety of cytokines (e.g., IL-1β, IL-1α, IL-6, GM-CSF, and TNF-α) and proliferate *in vitro* without the addition of exogenous cytokines and HGFs (99–101). In particular, IL-1β was shown to act as an autocrine growth factor for AML blasts by inducing the production of HGFs and cytokines, including GM-CSF and IL-6 (50, 59, 99, 102–105). Moreover, IL-1-stimulated AML blast cells secreted increased levels of TNF-α, which synergized with IL-3- or GM-CSF-induced AML cell proliferation and colony formation (106). Of note, increased autonomous and/or HGF- and cytokine-induced *in vitro* proliferation of patient-derived leukemic cells correlated with negative clinical outcomes, including lower complete remission rates, higher risk for relapse, and shorter AML patient survival (107–109). *In vivo*, however, LSC and blast growth, survival, and protection from therapeutic agents do not exclusively rely on endogenous cytokine signaling, but strongly depend on AML cell interactions with the leukemic BM microenvironment and the latter’s provisioning of supporting ligands and soluble factors, including cytokines (**Figure 1**), some of which are discussed in the following sections (110).

## AML supporting cytokines

### Osteopontin

Osteopontin, a secreted matrix glycoprotein produced by many cell types (e.g., stomal, endothelial, epithelial and immune cells), is crucial for the regulation and/or induction of inflammation, angiogenesis, proliferation, migration, and apoptosis throughout the body. During normal hematopoiesis, osteopontin is predominantly produced by osteoblasts within in the endosteal BM region, to guide and maintain healthy HSCs within supportive niches (111). Interestingly, PB and BM osteopontin levels were significantly increased in AML patients compared to healthy controls, and high osteopontin BM levels were associated with reduced overall and event-free survival (82, 83). Osteopontin was not, however, exclusively expressed by cells of the osteolineage within the leukemic BM, although it was strongly expressed by AML blasts (82). Additionally, it was shown that AML patient-derived mesenchymal stromal cells (MSCs) or healthy MSCs co-cultured with AML cells undergo osteogenic differentiation and produce increased amounts of osteopontin (112). Functionally, osteopontin was shown to upregulate AKT, mTOR, PTEN, and β-catenin mRNA expression in AML cells *in vitro* (113) and increase AML LSC self-renewal, proliferation, and expression of anti-apoptotic and cell-cycle-associated genes, thereby leading to accelerated disease progression in an MLL-AF9 driven AML mouse model (84). Direct targeting of osteopontin is difficult due to its ubiquitous expression and, so far, has been limited to approaches utilizing RNAi or blocking antibodies and aptamers in breast cancer models. Although delivered without specificity to cell type, these initial treatment studies confirm the antitumoral effect of osteopontin inhibition (114) and call for testing in AML disease models.

### Interleukin-1

Due to its pleiotropic effects, IL-1-mediated signaling is recognized as a central hub between inflammation and cancer, including leukemia development and progression (32, 115–119). In AML patients, multiple studies have reported increased levels of IL-1β and IL-1 receptors as well as decreased levels of interleukin-1 receptor antagonist (IL-1RA) in PB and BM (41, 42, 56, 120). In an MLL-AF9-driven leukemic mouse model, chronic exposure to IL-1β accelerated leukemia progression and impaired normal hematopoiesis by modulating stromal niche support. Using both *in vitro* and *in vivo* AML models it was shown that depletion or deletion of IL-1RA resulted in reduced expansion of AML progenitor cells and partially restored normal hematopoiesis (121). This was confirmed by targeting IL-1 receptor signaling *via* inhibition of p38 MAPK, which enabled normal HSPCs to expand in the presence of IL-1β (41). Of note, *in vivo* AML development was curbed by knockout of IL-1 receptor in the MLL-AF9 mouse model but was increased in FLT3-ITD-driven leukemic mice (122), suggesting different dependencies on IL-1 signaling. Additionally, it was shown that AML HSPCs express high levels of IL-1 receptor accessory protein (IL-1RAP), which contributed to increased IL-1β production, AML cell proliferation and survival, but reduced normal hematopoiesis. This phenotype was further promoted when co-culturing AML CD34^+^ HSPCs on MSCs (123, 124). Inhibition of IL-1RAP signaling antagonized this effect and enabled HSC proliferation in the presence of AML cell-conditioned media (124). In line with these AML cell-supporting functions, gene expression analysis revealed reduced overall survival (OS) of AML patients who expressed high levels of IL-1RAP (123). These observations suggest an important role for the IL-1β signaling pathway in the pathogenesis of AML and encourage studies to evaluate the therapeutic effects of targeting IL-1 signaling (117, 120). Multiple US Food and Drug Administration (FDA)-approved IL-1 blockers [Anakinra (Kineret); Rilonacept (Arcalyst); Canakinumab (Ilaris)] are already available. In particular, the effect of Canakinumab is being intensely evaluated in the CANTOS trail (NCT01327846), a randomized, double-blind, placebo-controlled phase 3 study involving 10,061 patients with solid tumors as well as hematological malignancies like chronic myelomonocytic leukemia (CMML) and myelodysplastic syndrome (MDS) (125, 126). However, additional studies will be required to fully understand the therapeutic value of targeting IL-1 and in particular IL-1β in hematological malignancies, including AML.

### Interleukin-6

IL-6 is a potent pro-inflammatory cytokine which is crucial for a rapid and coordinated immune response during infections and tissue injuries, but also helps to maintain the hematopoietic system (127–129). Deregulated expression of IL-6 is associated with inflammatory and autoimmune diseases as well as skewed hematopoiesis and leukemia (104, 127, 130). In AML patients with reduced OS, blood and BM serum levels of IL-6 are increased (39, 53, 131). Further studies confirm these findings and suggest that IL-6 levels correlate with poor prognosis, rapid disease progression, and resistance to chemotherapy (39, 132–134). The combined assessment of PB IL-6 and FLT3-ligand levels during AML induction therapy revealed that patients with persistent high IL‐6 levels display lower survival rates compared to patients with decreasing IL-6 and increasing FLT3-ligand levels (134). Similarly, low IL-6 levels accompanied by high IL-10 levels have been linked to better prognosis (39). Although AML blasts are clearly exposed to microenvironment-derived (135–137) and self-produced IL-6 (104), it is disputed how IL-6 contributes to AML progression. Curiously, all AML samples express the IL-6 receptor but only a subset responds to IL-6 treatment *in vitro* (48, 67, 69, 70, 131, 138). However, multiple studies suggest that IL-6-induced STAT3 signaling promotes AML by inducing chemoresistance (132, 135, 139). Hou and colleagues showed that BM MSCs promote chemoresistance against daunorubicin and cytosine arabinoside (Ara-c) by increasing IL-6 secretion and activation of STAT3 signaling and the oxidative phosphorylation metabolic pathway in AML cells (135). Zhang et al. showed that IL-6-induced STAT3 signaling promotes CD36 expression, CD36-mediated uptake of fatty acids, and chemoresistance against Ara-c (139). Several IL-6 or IL-6 receptor-blocking antibodies have demonstrated promising results in (pre-) clinical studies for the treatment of cancers, chronic inflammation, and autoimmune diseases (130). While Siltuximab (CNTO 328; IL-6-blocking antibody) has been proposed as a treatment option for myelodysplastic syndrome (MDS) and multiple myeloma and is FDA-approved for the treatment of idiopathic multicentric Castleman’s disease (140), Siltuximab in the AML setting has so far only been investigated in an AML xenograft mouse model that mimics end-stage BM failure. In that study, Siltuximab treatment antagonized AML-induced anemia and BM failure and prolonged mouse OS (141).

### Interleukin-8

IL-8 (CXCL8) belongs to the CXC family of chemokines and is best known for its role as a chemoattractant for neutrophils (142). While production of IL-8 can be induced by various stimuli, including lipopolysaccharide, IL-1, and TNF in healthy cells, many tumor cells express IL-8 constitutively (142) or in a hypoxia-, acidosis-, or chemotherapy-induced manner, leading to anti-apoptotic and growth-supporting MAPK, PI3K, FAK and SRC18 signaling (143). In AML, constitutive production of IL-8 has been observed in both AML cell lines and primary AML samples, together with expression of functional IL-8 receptors [IL-8RA (CXCR1) and IL-8RB (CXCR2)] (144–146). Interestingly, AML cell-derived IL-8 also signals in a paracrine manner and affects neighboring non-leukemic cells in the BM microenvironment. Hypoxia-induced IL-8 secretion by AML cells resulted in increased migration of MSCs into the leukemic BM niche (147). MSCs, in turn, prevent apoptosis and confer drug resistance on leukemic cells by up-regulation of anti-apoptotic proteins and secretion of growth factors, cytokines, and extracellular vesicles (148, 149). Importantly, it has been shown that IL-8 production and secretion by MSCs, fibroblasts, and endothelial cells is induced or increased upon their co-culture with AML cells, thereby contributing to reduced apoptosis and increased proliferation and chemoresistance of the AML cells (150–152). In patients, this bidirectional signaling seems to result in elevated IL-8 levels in PB and BM levels (72), which additionally might contribute to impaired neutrophil migration and hematopoiesis (153). However, further confirmation, especially in the context of AML, is required. Inhibition of the IL-8–IL-8R axis has been proposed as a novel therapeutic intervention targeting the aberrant leukemic BM microenvironment. Blocking the IL-8 pathway with neutralizing antibodies has been shown to restore the sensitivity of malignant cells to chemotherapeutics and reduce AML cell proliferation (150, 151). Using knockdown or pharmacological inhibition approaches, Schinke and colleagues showed that inhibition of IL-8RB-mediated signaling leads to a significant reduction in proliferation and G0/G1 cell cycle arrest in several leukemic cell lines and primary MDS/AML samples (119).

### CXC motif chemokine 12

The chemotactic cytokine (chemokine) CXCL12, also referred to as SDF-1, is secreted by a variety of cells including stromal cells, fibroblasts, and epithelial cells (154). CXCL12 initiates signaling by binding to its receptors CXCR4 and CXCR7 and plays a crucial role in regulating hematopoiesis (proliferation, differentiation, survival) and hematopoietic cell trafficking to and within the BM (95, 155), but also contributes to tumor growth, survival, metastasis, vascularization, and chemoresistance of several types of cancer (95, 156–160). In AML, low expression of CXCL12, high expression of CXCR4 and low to intermediate expression of CXCR7 have been measured on AML blasts in comparison to normal HSPCs (92–94). Interestingly, decreased CXCL12 and increased CXCR4 expression by AML blasts was associated with reduced patient relapse-free and overall survival OS (161–164). Within the healthy BM, CXCL12 is mainly secreted by perivascular stromal cells [mesenchymal stem and CXCL12-abundant reticular (CAR) cells], endothelial cells, and osteoblasts, thereby guiding, retaining, and regulating HSPCs to and within supportive BM niches (16). Within the AML BM microenvironment, it has not yet been determined which and to what extent cell populations produce and secrete CXCL12. *In vitro*, CXCL12 was shown to promote AML cell growth, survival, and chemoresistance (95–98) by activating or inducing the pro-survival proteins PI3K/AKT, MAP3K/ERK1/2, MYC, Bcl-2, and Bcl-XL (93, 96, 165). *In vivo*, however, while the deletion of CXCR4 in AML MLL-AF9^+^ HSPCs prolonged leukemic mouse survival, deletion of CXCL12 within the AML microenvironment did not alter the development and progression of the disease (166). This surprising finding suggests that CXCR4 signaling can support AML cells in a CXCL12-independent manner. Nevertheless, blocking the CXCL12/CXCR4 axis represents an attractive therapeutic strategy and several CXCR4 and CXCL12 inhibitors have been developed and used in preclinical and clinical models to induce the mobilization of the AML cells from the BM into the circulation with the aim of increasing their exposure to chemotherapeutic agents (95, 167). Plerixaflor (NCT01319864, NCT01352650, NCT01027923), LY2510924 (NCT02652871), and PF-06747143 (NCT02954653) are among the antagonists that have been under Phase 1 clinical trials to test for safety, tolerability and clinical activity, either alone or in combination with standard chemotherapy in AML patients.

## AML inhibiting cytokines

### Interferon-γ

Interferon-γ (IFN-γ) is one of the lead cytokines of cellular immunity. It is mainly secreted by activated lymphocytes (168) and orchestrates tumor defense by regulating AML blast survival and apoptosis (118). While T cells obtained from AML patients at primary diagnosis exhibit increased IFN-γ production, strongly reduced levels of IFN-γ were observed in CD8^+^ T cells from patients who developed relapsed AML after allogeneic HCT (allo-HCT), whereas patients without relapse did not show reduced IFN-γ production (169). This suggests that lower IFN-γ levels may elevate the risk of relapse. An early phase 1 trial was recently started to evaluate the potential of IFN-γ treatment in AML patients with reoccurring disease after allo-HCT (NCT04628338). However, manipulation of IFN-γ levels in AML patients should be carefully assessed, because systemic administration of IFN-γ is limited by rapid IFN-γ clearance and insufficient distribution to tumor sites. Moreover, while IFN-γ can restore T cell-mediated anti-cancer immunity and the surface expression of HLA class II molecules, the loss of which has been shown to impair AML recognition by donor T cells (170), IFN-γ is also capable of promoting PD-L1 and PD-L2 expression in AML (171, 172). Indeed, high expression of PD-L1 and PD-L2 is associated with poor OS in AML patients (173, 174). Binding of PD-L1/PD-L2 to the receptor PD-1 increases T cell exhaustion, promotes effector T cell apoptosis, induces the resistance to effector T cell-mediated killing (175) and increases the conversion and development of Tregs which have strong immune-suppressive abilities (176). Thus, the potential induction of PD-L1 and PD-L2 by IFN-γ may have unfavorable consequences, because the PD-1/PD-L1/PD-L2 axis helps the tumor to maintain an immunosuppressive microenvironment, thereby promoting immune evasion and survival of cancer cells (177). In addition to T cells, innate lymphoid cells type I (ILC1s) are another potent source of IFN-γ in healthy individuals. ILCs are important players of innate immune responses by reacting promptly to signals, or inducer cytokines, expressed by tissue-resident cells. ILC1s function as a first line of defense against intracellular pathogens, such as viruses, and tumors (178). By secreting IFN-γ, healthy ILC1s induce apoptosis and block differentiation by modulating JAK-STAT or PI3K/AKT signaling. However, in AML, ILC1s exhibit reduced IFN-γ secretion and lose their ability to suppress the development of LSCs and antagonize AML progression (179). ILC1s thus seem pivotal as an anti-cancer immune cell, and administration of *ex vivo*-expanded ILC1s could provide a new immunotherapeutic approach to ensure that IFN-y levels are locally increased within leukemic niches. Importantly, this approach would significantly decrease toxicity for AML patients in comparison to systemic delivery of IFN-y (179).

### Interleukin-4

IL-4 is a signature cytokine of type II inflammation and regulates many aspects of Th2-mediated immunity (180). In epithelial cancers, IL-4 is generally considered to have pro-tumorigenic and pro-metastatic functions, suggesting that inhibition of the IL-4/IL-4R axis may be beneficial to limiting diseases (181, 182). Yet, in hematological cancers, a tumor-promoting role of IL-4 is controversial. Already in the early 1990s there were studies reporting that IL-4 might also have tumor-limiting functions, by suppressing IL-1-induced proliferation of AML cells (60, 64, 65). More recent findings substantiate those earlier observations and show that IL-4 has the potential to inhibit survival of AML cell lines as well as patient-derived AML cells, irrespective of their cytogenetic status and French-American-British (FAB) subtype, without affecting normal HSPCs. Anti-leukemic effects of IL-4 are at least partially dependent on STAT6 and Caspase-3, which agrees with the crucial role of STAT6 in mediating IL-4’s effects downstream of the IL-4 receptor (183). In addition, IL-4-induced STAT6, in cooperation with the nuclear receptor protein proliferator-activated receptor gamma (PPARγ), upregulates the expression of prostaglandins. In particular, COX (cyclooxygenase)-dependent prostaglandins, so-called CyPGs, play an important role in apoptosis (184). After stimulation by IL-4, these lipid mediators are increasingly produced *via* the COX/prostaglandin axis, which leads to activation of p53 and caspase-3 and subsequently stimulates apoptosis of leukemic cells (185). The fact that IL-4 treatment specifically acts on AML blasts, but does not affect HSCs, even upon long-term treatment, makes IL-4 an interesting candidate for therapeutic intervention in AML. Yet, despite its promising role as an anti-leukemic cytokine, IL-4 additionally promotes the differentiation of immune cells, including M2 macrophages, which are regarded as having a leukemia-supporting phenotype. M2 macrophages release various cytokines and growth factors that promote blast survival and proliferation, induce proangiogenic effects and can directly inhibit CD8^+^ T cell-mediated killing of blast cells (186, 187). Therefore, more detailed studies are required to assess the value of IL-4 as an antileukemic molecule.

### Interleukin-10

IL-10, an anti-inflammatory cytokine produced by several immune cells, is crucial for limiting immune responses and damage caused by long-lasting inflammation (188). In AML patients, significantly higher levels of plasma IL-10 are observed (53, 56, 73) which directly correlate with prolonged overall patient survival, event-free survival and higher complete remission rates (39, 189, 190). *In vitro*, IL-10 treatment of AML blasts inhibited spontaneous AML blast proliferation and colony formation by negatively affecting the production and secretion of pro-leukemic cytokines (IL-1α, IL-1β, TNF-α, GM-CSF, GM-CSF, and IL-6) (74–76, 191). However, contrary to these findings, there is also evidence that IL-10 together with IL-35 — the latter a CD4^+^ and CD8^+^ T cell-suppressing and T regulatory cell (Treg)-supporting cytokine upregulated in AML (118) — promotes AML cell proliferation, survival and chemoresistance. So far, ICOS1^+^ and PD1^+^ Treg cells as well as BM-MSCs have been suggested as a source for IL-10 in the AML microenvironment (176, 192), contributing to the establishment of an IL-10-induced immunosuppressive and anti-inflammatory niche which ensures LSC survival and stemness (193, 194). Therefore, despite the direct correlation between IL-10 serum level and prolonged patient survival and treatment response (39, 189), antagonizing IL-10 signaling could support current chemotherapeutic approaches to irradicate LSCs and decrease the patient relapse rate (195). However, so far, no combinatory studies have been performed. Interestingly, Chen et al. recently highlighted the IL-10 receptor as a potential candidate for AML immunotherapy as it is significantly upregulated on AML cells in patients and is required for LSC stemness. CAR-T cells harboring an IL-10 peptide structure within their antigen-binding domain were shown to recognize and bind to the IL-10 receptor of multiple AML cell lines (MV4-11, Kasumi-1, U937, THP-1 and MOLM-13) and primary AML cells, thereby inducing the CAR T-cell mediated killing of these cells *in vitro* and *in vivo* (196). Although the function of IL-10 may be patient-dependent, immunomodulatory agents that block IL-10 could offer an interesting approach for treatment of AML.

### Interleukin-12p70: a new trick for an old cytokine

More than a decade ago, IL-12p70 was shown to inhibit the angiogenic potential but not the survival or proliferation of AML cells (77) and to increase T-cell proliferation and cytotoxicity against leukemic cells *in vitro* (78, 79). Multiple *in vitro* co-culture studies overexpressing IL-12p70 in dendritic cells have confirmed these findings (197, 198) and paved the way for initial immunotherapies using genetically modified dendritic cells (phase 1 clinical trial NCT01734304) (199), thereby trying to avoid the toxicity of systemic administration of IL-12. Recently, another elegant therapeutic approach based on transplantation of genetically modified AML blasts constitutively expressing IL-12 in a vaccine-like manner was successfully established in murine and human cells (197) and is currently under investigation in a phase 1 clinical trial (NCT02483312).

## Conclusion and future perspectives

Despite intensive research leading to new and targeted therapeutic strategies for AML in recent years, the prognosis for a large proportion of patients remains poor. Due to the highly heterogeneous nature of AML, current therapies often only eliminate specific subclones but cannot permanently halt disease progression. As in many other cancers, chronic inflammation, characterized by the release of pro-inflammatory cytokines and growth factors, which significantly influence the interaction of tumor and immune cells in the tumor microenvironment, can also be observed in AML. On the one hand these cytokines may ensure survival of cancer cells by promoting tumor cell proliferation while inhibiting the antitumor immune responses; on the other hand, some cytokines contribute to cancer cell elimination by supporting the body’s own immunological defense mechanisms. Therapeutic application of cytokines, or therapies that specifically target cytokines and/or their receptors, may provide new avenues for the treatment of AML patients in the coming years. In particular, IL-1β, IL-6 and CXCL12 might be promising new druggable targets. However, due to the pleiotropic effects of most cytokines, which control both tumor growth and anti-tumor immune responses, we are faced with the challenge of establishing new therapies, which on the one hand inhibit tumor growth and at the same time specifically enhance the anti-tumor response. The use of combination therapies, in which potential inhibitors of inflammatory cytokines are combined with other therapeutic molecules and agents, may prove promising for this purpose.

## Author contributions

Conceptualization, JH-H, ML, PWK, writing—original draft preparation, ML and PWK, writing—review and editing, JH-H, visualization, ML and funding acquisition, JH-H. All authors contributed to the article and approved the submitted version.

## Funding

This work was supported by the County of Salzburg, Cancer Cluster Salzburg [grant number 20102-P1601064-FPR01-2017], the Austrian Science Fund (FWF) [grant numbers W1213 and P33969], the Biomed Center Salzburg (project 20102-F1901165-KZP), the European Interreg project EPIC (grant number ITAT1054), and by the Priority program ACBN, University of Salzburg.

## Conflict of interest

The authors declare that the research was conducted in the absence of any commercial or financial relationships that could be construed as a potential conflict of interest.

## Publisher’s note

All claims expressed in this article are solely those of the authors and do not necessarily represent those of their affiliated organizations, or those of the publisher, the editors and the reviewers. Any product that may be evaluated in this article, or claim that may be made by its manufacturer, is not guaranteed or endorsed by the publisher.
